# Lineage-Associated Genomic and Phenotypic Features of Bovine Mastitis-Associated *Staphylococcus aureus* from Northwestern China

**DOI:** 10.3390/microorganisms14081698

**Published:** 2026-08-02

**Authors:** Ting Zhou, Xinying Tao, Zhiying Xue, Yongwang Huang, Yueqi Dong, Mohan Liu, Yilun Hu, Xin Wang, Yan Cui

**Affiliations:** 1 College of Veterinary Medicine, Gansu Agricultural University, Lanzhou 730070, China; zhouting@nwafu.edu.cn; 2 College of Veterinary Medicine, Northwest A&F University, Yangling 712100, China; xzy221106@nwafu.edu.cn (Z.X.); huang4071@nwsuaf.edu.cn (Y.H.); dyq771213@nwafu.edu.cn (Y.D.); lmhc929@nwafu.edu.cn (M.L.); huyilun0409@nwafu.edu.cn (Y.H.); 3 Department of Quality Management, Inspection and Testing, Yibin University, Yibin 644000, China; taoxinying2024@sina.com; 4 College of Food Science and Engineering, Northwest A&F University, Yangling 712100, China; xinwang7516@nwsuaf.edu.cn

**Keywords:** bovine mastitis, *Staphylococcus aureus*, whole-genome sequencing, molecular epidemiology, antimicrobial resistance, biofilm formation, virulence genes

## Abstract

*Staphylococcus aureus* is a major contagious pathogen causing bovine mastitis, with persistence and antimicrobial resistance posing ongoing challenges in dairy herds. From July 2022 to July 2023, 942 mastitis milk samples (267 clinical, 675 subclinical) from 10 farms in northwestern China yielded 98 *S. aureus* isolates (10.4%), which were characterized by whole-genome sequencing, antimicrobial susceptibility testing, biofilm assays, and virulence profiling. Ten sequence types, seven clonal complexes, and 16 spa types were identified, dominated by ST1 (42.9%) and ST97 (24.5%). ST1 was mainly associated with subclinical mastitis, whereas ST97 was enriched in clinical cases; a novel ST9955 (13.3%) was exclusively detected in subclinical isolates. No methicillin-resistant *S. aureus* was detected. Resistance was most frequently observed to penicillin (42.9%), erythromycin (28.6%), clindamycin (22.4%), and tetracycline (10.2%), with higher rates in clinical isolates. 65.3% of isolates exhibited strong biofilm-forming ability, which was significantly more frequent in subclinical cases (70.3% vs. 55.9%, *p* < 0.05). Virulence genes showed conserved adhesion/biofilm determinants but lineage-dependent distribution of immune evasion and toxin genes, with stable co-occurrence among capsule, biofilm, T7SS, iron acquisition, and immune evasion loci. Overall, the population was diverse but strongly lineage-structured, dominated by a few successful clones, highlighting the value of lineage-resolved surveillance for understanding and controlling bovine mastitis.

## 1. Introduction

Bovine mastitis remains a major challenge to intensive dairy production, leading to reduced milk yield and quality, increased treatment and culling costs, impaired animal welfare, and potential risks to dairy safety [1,2]. The widespread use of antimicrobial agents and the persistent circulation of pathogens within dairy herds have contributed to the emergence of antimicrobial resistance and raised public health concerns [2,3]. Among the causative agents, *Staphylococcus aureus* (*S. aureus*) is one of the most important contagious pathogens associated with bovine mastitis and can cause both clinical mastitis (CM) and subclinical mastitis (SCM) [2,4]. Compared with many environmental mastitis pathogens, *S. aureus* exhibits strong host adaptation, efficient cow-to-cow transmission, and the ability to establish persistent intramammary infections, making it a major obstacle to effective mastitis control [3,4]. Its colonization of mammary tissue, teat skin, and milking-related surfaces facilitates long-term maintenance and dissemination within dairy herds [2,4]. Therefore, understanding the bacterial characteristics underlying persistence and infection-associated phenotypic variation is essential for developing targeted surveillance and control strategies.

The persistence of *S. aureus* within dairy herds is mediated by a combination of bacterial traits that promote colonization, survival, and transmission. Surface-associated adhesins facilitate attachment to mammary epithelial cells and extracellular matrix components, while invasion-associated mechanisms may enable bacterial persistence within host tissues and reduce immune-mediated clearance [2,5,6]. Biofilm formation further enhances bacterial survival by increasing tolerance to host immune defenses and antimicrobial treatment, thereby contributing to chronic or recurrent intramammary infection [7,8,9]. In addition, capsule biosynthesis, iron acquisition systems, and other virulence-associated factors promote immune evasion, nutrient acquisition, and tissue damage, enhancing bacterial fitness within the mammary gland environment [2,10,11].

Persistence-associated characteristics are not uniformly distributed among bovine *S. aureus* isolates, but vary with genetic background and clonal lineage [12,13,14]. Molecular epidemiological and comparative genomic studies have identified several host-adapted lineages within bovine *S. aureus* populations [13,15]. Among them, CC97, CC151, CC705, and CC133 are commonly reported as major bovine-associated lineages, whereas CC1 and CC398 have also been detected in dairy cattle populations [13,14,16]. Comparative analyses further indicate that these lineages differ in virulence gene repertoires, mobile genetic elements, host-adaptation features, and interactions with the bovine immune system [13,16,17]. These lineage-associated differences indicate that bovine *S. aureus* populations are genetically diverse and structured into distinct host-adapted lineages [13,15]. However, the distribution and phenotypic characteristics of these lineages may differ across geographic regions and dairy production systems, suggesting that regional genomic surveillance combined with phenotypic characterization is important for understanding lineage-specific adaptation and pathogenic potential [13,14].

Whole-genome sequencing (WGS) has become an important tool for characterizing population structure, host adaptation, antimicrobial resistance determinants, virulence-associated loci, and mobile genetic elements in *S. aureus* [13,16]. When combined with antimicrobial susceptibility testing, biofilm phenotyping, and virulence profiling, WGS enables comprehensive characterization of lineage-associated traits and facilitates investigation of the genetic basis underlying persistence, adaptation, and pathogenicity [13,15,18]. Such integrated genomic and phenotypic analyses facilitate investigation of the epidemiology and adaptive evolution of bovine *S. aureus* populations beyond that achievable through conventional phenotypic characterization alone [13,15].

Genomic and molecular epidemiological investigations of bovine *S. aureus* in China have revealed considerable genetic diversity among mastitis-associated isolates. These studies have provided valuable information regarding the prevalence, antimicrobial resistance profiles, molecular types, and virulence characteristics of bovine *S. aureus* isolates from different regions [19,20,21,22,23,24]. However, these investigations have generally focused on individual regions and have primarily characterized molecular types, antimicrobial resistance, virulence genes, or biofilm-related traits [19,20,21,22,23,24]. Studies integrating genomic characterization with infection-associated phenotypes remain limited, particularly across different mastitis presentations and geographic regions. Ningxia, Gansu, Shaanxi, and Inner Mongolia are located in northwestern China and adjacent dairy-producing areas, where the spatial aggregation of herbivorous animal husbandry, intensive management, mechanization, and animal movement may increase the risk of pathogen persistence and dissemination within and between farms [22,25,26]. Comparative genomic and phenotypic characterization of bovine *S. aureus* from these regions is therefore needed to improve understanding of regional population structure and infection-associated characteristics.

To address this knowledge gap, we conducted an integrated genomic and phenotypic investigation of *S. aureus* isolates recovered from CM and SCM cases in large-scale dairy farms across northwestern China. The study aimed to characterize the dominant clonal lineages and examine their associations with antimicrobial resistance, biofilm-forming capacity, and virulence gene composition.

## 2. Materials and Methods

### 2.1. Sample Collection

**Figure 1:**
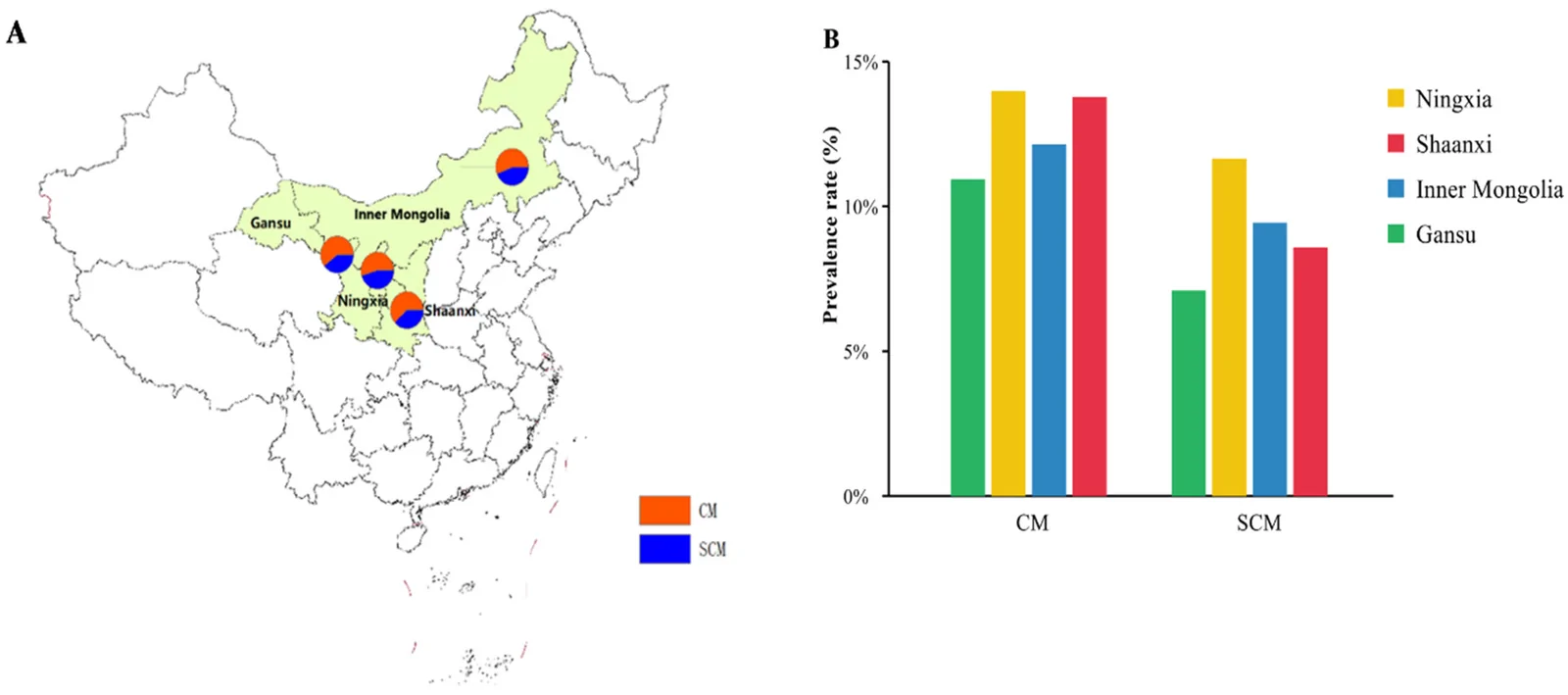
Geographic distribution and prevalence of clinical mastitis (CM) and subclinical mastitis (SCM) in four regions of China. (**A**) Sampling regions and proportional distribution of CM and SCM cases in Gansu, Ningxia, Inner Mongolia, and Shaanxi. Orange indicates CM and blue indicates SCM. (**B**) Prevalence of *S. aureus* in CM and SCM milk samples. No significant differences were observed among regions within the CM or SCM groups by Pearson’s chi-square test (*p* > 0.05).

A total of 942 milk samples were collected between July 2022 and July 2023 from Holstein dairy cows, mainly multiparous cows in their second to third parity, on 10 commercial dairy farms across four regions in northwestern China, including Gansu (*n* = 247, three farms), Ningxia (*n* = 299, two farms), Shaanxi (*n* = 192, three farms), and Inner Mongolia (*n* = 204, two farms) (Figure 1A). Of these, 675 samples were obtained from cases of subclinical mastitis and 267 from clinical mastitis. SCM was defined as a somatic cell count (SCC) ≥ 2 × 10^5^ cells/mL and/or a positive California Mastitis Test (CMT) result [27]. In contrast, CM was diagnosed based on visible milk abnormalities (e.g., clots or discoloration), localized udder inflammation (e.g., swelling, heat, or pain), and systemic clinical symptoms, as previously described [28]. Farm-level information was obtained from farm records and interviews with farm veterinarians. Antimicrobials reported to be used for mastitis treatment included penicillin, streptomycin, gentamicin, cephalosporins, and lincosamides. All sampled farms used mechanical milking systems and routine teat disinfection before milking.

For SCM cases, composite milk samples were collected from all four udder quarters of each cow, whereas for CM cases, samples were collected only from the clinically affected quarter. All samples were collected aseptically into sterile 50 mL polypropylene centrifuge tubes. Before sampling, udders were cleaned, dried, and teat ends were disinfected with 75% ethanol. After discarding the first three milk streams, approximately 20–25 mL of milk was collected into each tube. Samples were immediately placed in insulated containers with ice packs and transported under a dry ice cold chain to the microbiology laboratory at Northwest A&F University (Yangling, China) for *S. aureus* isolation and identification.

### 2.2. Bacterial Isolation and Identification

A 100 μL aliquot of each milk sample obtained from SCM and CM cases was inoculated onto WZ-*S. aureus* chromogenic agar plates, a selective medium developed by our laboratory for the isolation of *S. aureus*. The plates were incubated at 37 °C for 24 h. Subsequently, two to four presumptive colonies from each plate were then subcultured onto tryptic soy agar (TSA) (Hope Bio, Qingdao, China) plates for purification and incubated at 37 °C for an additional 24 h. Purified strains were preserved in sterile Luria–Bertani (LB) (Hope Bio, Qingdao, China) broth containing 30% glycerol at −80 °C until further analysis. Presumptive *S. aureus* strains were initially identified by matrix-assisted laser desorption/ionization time-of-flight mass spectrometry (MALDI-TOF MS, Bruker, Bremen, Germany).

### 2.3. DNA Extraction

The 98 confirmed *S. aureus* isolates were streaked onto TSA plates and incubated at 37 °C for 24 h. A single colony was then inoculated into tryptic soy broth (TSB) (Hope Bio, Qingdao, China) and cultured overnight at 37 °C with shaking at 180 rpm. Subsequently, 2 mL of the overnight culture was transferred to a sterile microcentrifuge tube, centrifuged at 12,000 rpm for 5 min, and the supernatant was removed. Genomic DNA was extracted using the SteadyPure Bacterial Genomic DNA Extraction Kit (Accurate Biotechnology (Hunan) Co., Ltd., Changsha, China) according to the manufacturer’s instructions.

### 2.4. Whole-Genome Sequencing and Analysis

DNA samples from the 98 confirmed *S. aureus* isolates, with concentrations of 50–100 ng/μL, were submitted to Tianjin BioNova Biotechnology Co., Ltd. (Tianjin, China), and whole-genome sequencing was performed on the Illumina NovaSeq platform. Raw sequencing reads were quality-filtered using fastp v0.23.2 to remove adapter sequences and low-quality reads [29], and the resulting clean reads were de novo assembled with SPAdes v3.15.5 [30]. Multilocus sequence typing (MLST) was assigned using the PubMLST database (https://pubmlst.org/; accessed 10 October 2025) [31], while spa typing was performed using the online spaTyper tool (https://cge.food.dtu.dk/services/spaTyper/; accessed 15 October 2025) [32]. Related sequence types (STs) were assigned to corresponding clonal complexes (CCs) using BioNumerics v8.1.

Phylogenetic relationships were inferred based on core genome SNPs. Raw reads were mapped to the reference genome using Snippy v4.6.0 to generate a core SNP alignment, followed by the identification and removal of putative recombinant regions using Gubbins v3.4.13. A maximum-likelihood phylogenetic tree was then constructed from the filtered core SNP dataset using IQ-TREE v2.2.0, with visualization and annotation performed in ChiPlot (https://www.chiplot.online/; accessed 15 November 2025) [33].

Virulence genes were annotated against the Virulence Factor Database (VFDB) and categorized according to functional class [34]. Antimicrobial resistance genes were screened against the ResFinder database [35], and genotype–phenotype concordance was evaluated by comparison with antimicrobial susceptibility profiles.

### 2.5. Antimicrobial Susceptibility Testing

Antimicrobial susceptibility testing was performed for 14 antimicrobial agents. Susceptibility to penicillin (PEN), cefoxitin (FOX), oxacillin (OXA), tetracycline (TET), amikacin (AMK), trimethoprim–sulfamethoxazole (TMP–SMX), gentamicin (GEN), linezolid (LZD), chloramphenicol (CHL), clindamycin (CLI), erythromycin (ERY), ciprofloxacin (CIP), and rifampicin (RIF) was assessed by the disk diffusion method on Mueller–Hinton (MH) agar plates. The minimum inhibitory concentration (MIC) of vancomycin (VAN) was determined by the broth microdilution method. All testing procedures and interpretive criteria followed the Clinical and Laboratory Standards Institute (CLSI M100 34th ed) guidelines. *S. aureus* ATCC 25923 and ATCC 29213 were used as quality control strains for the disk diffusion and MIC assays, respectively. All assays were performed in three independent experiments.

### 2.6. Biofilm Formation Assays

Biofilm formation was assessed using the Congo red agar method as previously described by Singh et al. [36]. Based on colony morphology and color, isolates were classified as strong biofilm producers (black, rough colonies), moderate biofilm producers (dark intermediate colonies), or non-biofilm producers (red, smooth colonies).

Microtiter plate (MTP) assay: Biofilm formation was also assessed using the crystal violet-based semiquantitative microtiter plate method as previously described [37]. Assays were performed in sterile 96-well microplates. Following incubation, adherent biofilms were stained with crystal violet, and the bound dye was solubilized for quantification. Optical density was measured at 570 nm (OD_570_) using a microplate reader. Each isolate was tested in three technical replicates, and the assay was independently repeated three times. The cutoff value (ODc) was defined as the mean OD_570_ of the negative control plus three standard deviations. Biofilm production was categorized as follows: OD_570_ ≤ ODc, non-biofilm producer; ODc < OD_570_ ≤ 2 × ODc, weak biofilm producer; 2 × ODc < OD_570_ ≤ 4 × ODc, moderate biofilm producer; and OD_570_ > 4 × ODc, strong biofilm producer.

### 2.7. Statistical Analysis

Pearson’s chi-square test or Fisher’s exact test was used to compare *S. aureus* detection rates, antimicrobial resistance rates, and resistance gene carriage rates across geographic regions, mastitis types, and genotypic groups using GraphPad Prism v8.0.2 (GraphPad Software, San Diego, CA, USA). A two-sided *p* < 0.05 was considered statistically significant.

## 3. Results

### 3.1. Prevalence of S. aureus in Clinical and Subclinical Bovine Mastitis

Among the 942 milk samples analyzed, *S. aureus* was recovered from 98 samples, yielding an overall prevalence of 10.40% (98/942) (Table 1). The prevalence of *S. aureus* was higher in CM than in SCM samples (12.73% vs. 9.48%), although the difference was not statistically significant (χ^2^ = 2.17, *p* = 0.141). Across the four regions, overall prevalence ranged from 8.10% to 12.04%, with no significant regional differences observed (χ^2^ = 2.33, *p* = 0.507). When CM and SCM samples were analyzed separately, no significant regional differences were detected in either group (Figure 1B; all *p* > 0.05).

### 3.2. Genetic Diversity and Population Structure of S. aureus from Bovine Mastitis

**Figure 2:**
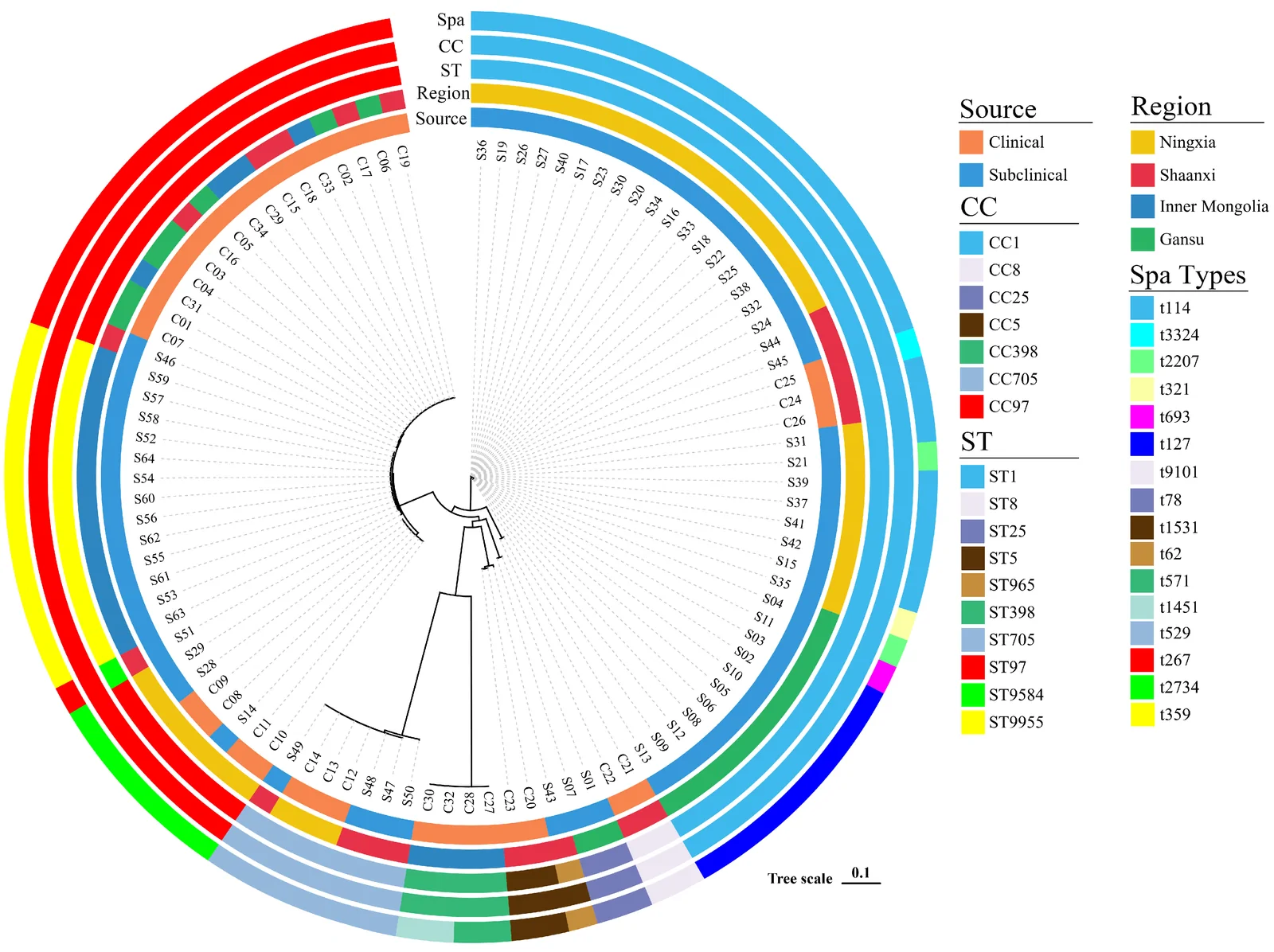
Core-genome phylogenetic structure of 98 bovine mastitis-associated *S. aureus* isolates from Northwest China. The phylogenetic tree was constructed based on core-genome SNPs. Colored rings from the inside to the outside represent isolate source, geographical region, sequence type (ST), clonal complex (CC), and spa type, respectively.

Core genome SNP-based phylogenetic analysis revealed substantial genetic diversity among the 98 *S. aureus* isolates recovered from bovine mastitis cases (Figure 2). These isolates were classified into seven clonal complexes (CCs), ten sequence types (STs), and sixteen spa types. CC1 and CC97 were the dominant clonal complexes, together accounting for 81.64% of all isolates, whereas CC705, CC398, CC5, CC8, and CC25 were detected at low frequencies. At the ST level, ST1 (42.86%) and ST97 (24.49%) remained the predominant sequence types, followed by the newly identified ST9955 (13.27%) and ST705 (7.14%). The remaining STs, including ST398, ST5, ST8, ST25, ST9584 and ST965, were infrequently detected. Among spa types, t114 (28.57%) and t267 (18.37%) were the most prevalent. spa typing also showed lineage-specific patterns, with ST1 encompassing multiple spa types, including t114 and t127, whereas ST97 was primarily associated with t267 and t2734. CM- and SCM-derived isolates were interspersed throughout the phylogeny rather than forming distinct source-specific clusters. Similarly, isolates from different geographic regions were distributed across multiple branches, although limited clustering was observed within some dominant lineages.

**Figure 3:**
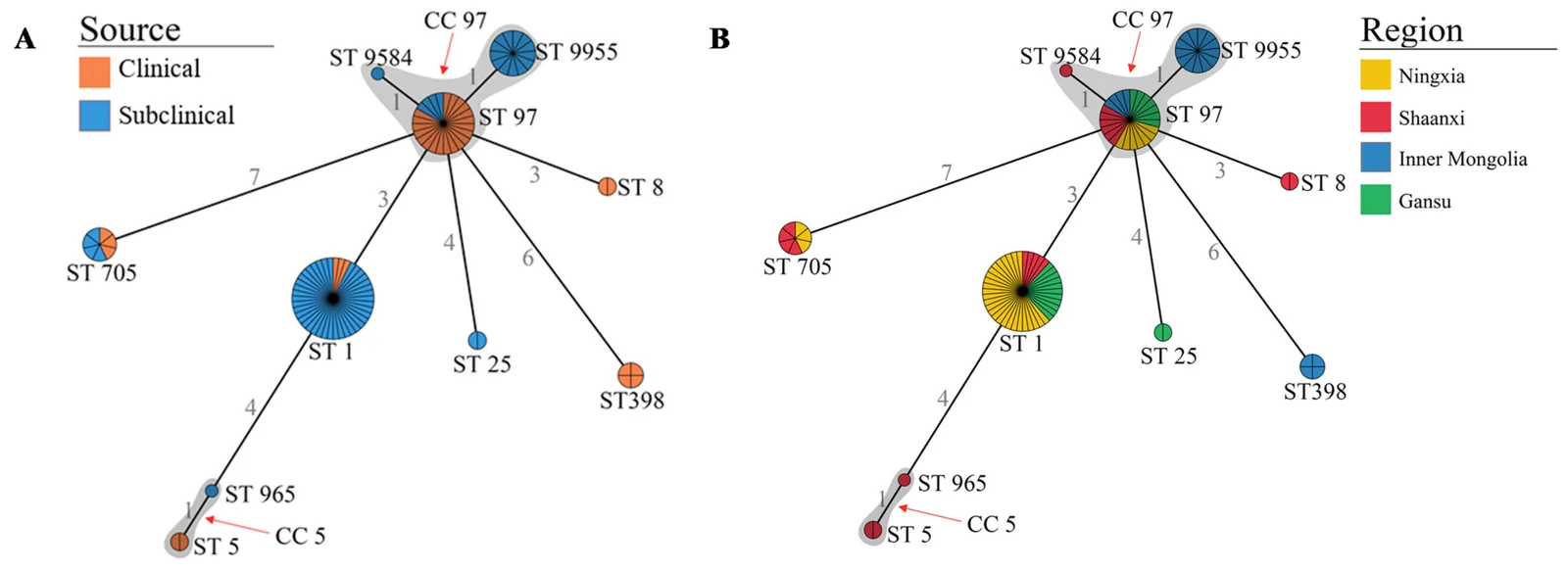
Minimum spanning tree of sequence types among 98 bovine mastitis-associated *S. aureus* isolates. Each circle represents a sequence type (ST), and the size of each circle is proportional to the number of isolates. The colors within circles indicate the distribution of isolates according to mastitis type (**A**) and geographic region (**B**). Numbers on connecting lines indicate allelic differences between STs. Clonal complexes (CCs) are indicated by grey shading and arrows.

The minimum spanning tree further illustrated the genetic relationships among the identified STs (Figure 3). ST1 and ST97 formed the two major central nodes, representing the dominant lineages within the study population. Several STs, including ST9955, ST705, ST398, ST8, ST25, and ST9584, were positioned close to ST97 in the minimum spanning tree, although they were assigned to different clonal complexes. ST5 and ST965 formed a smaller separate branch within CC5.

Marked differences in ST distribution were observed between CM and SCM isolates. ST1 was predominantly associated with SCM (39/42, 92.86%), whereas ST97 was mainly recovered from CM cases (20/24, 83.33%). Notably, the newly identified ST9955 was exclusively detected in SCM samples (13/13, 100%), while ST398 was confined to CM samples (4/4, 100%). The distributions of ST1, ST97, ST9955, and ST398 differed significantly between CM and SCM isolates by Fisher’s exact test. (Table 2) Although ST5 and ST8 were also detected exclusively in CM samples, these associations were not statistically significant, likely due to the small sample size.

No strict geographic clustering was observed across the four study regions, as isolates from Ningxia, Shaanxi, Inner Mongolia, and Gansu were distributed among multiple STs. ST1 was mainly represented by isolates from Ningxia and Gansu, whereas ST97 showed a broader geographic distribution across all four regions. In contrast, ST9955 displayed clear regional clustering, being detected exclusively in Inner Mongolia and only among SCM isolates. Overall, bovine mastitis-associated *S. aureus* in northwestern China showed considerable molecular diversity but was dominated by the ST1- and ST97-associated lineages, with distinct distribution patterns according to mastitis type and, to a lesser extent, geographic origin.

### 3.3. Accessory Genome Presence/Absence Patterns Among Major Sequence Types

**Figure 4:**
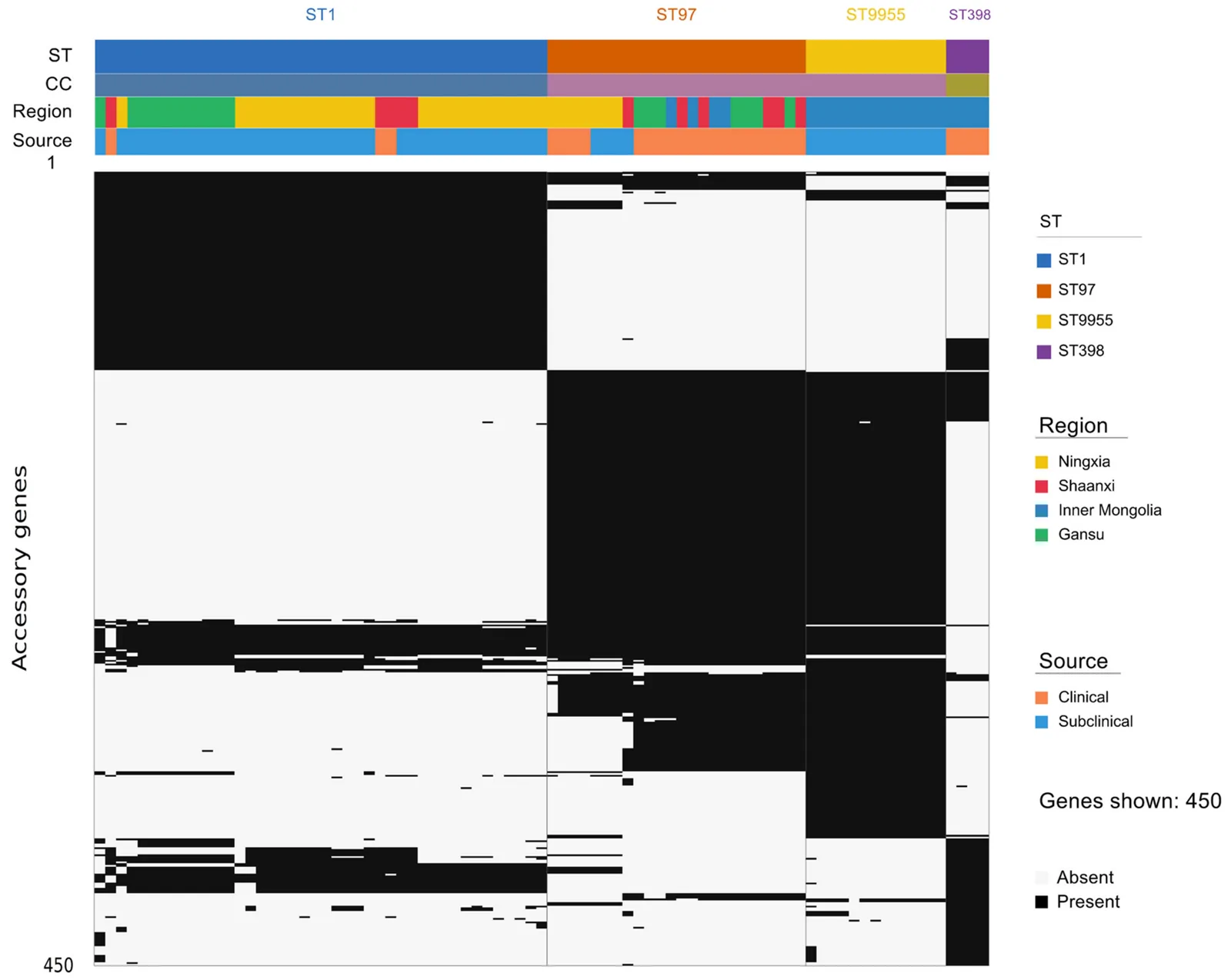
Accessory genome clustering of major sequence types among bovine mastitis-associated *S. aureus* isolates. The heatmap was generated from the accessory gene presence/absence matrix derived from Roary pan-genome analysis. A total of 450 variably distributed accessory genes were included for visualization. Columns represent individual isolates belonging to ST1, ST97, ST9955, and ST398, with annotation tracks indicating sequence type (ST), clonal complex (CC), geographic region, and clinical source. Black and light grey indicate gene presence and absence, respectively. CM, clinical mastitis; SCM, subclinical mastitis; ST, sequence type; CC, clonal complex.

To further characterize accessory genome variation among the major *S. aureus* sequence types, a heatmap was generated from the accessory gene presence/absence matrix derived from Roary pan-genome analysis, with 450 representative accessory genes selected for visualization (Figure 4). Distinct sequence type-associated clustering patterns were observed among ST1, ST97, ST9955, and ST398 isolates.ST1 isolates shared a characteristic accessory gene distribution pattern, with a set of accessory genes consistently detected within this lineage but less frequently identified in other sequence types. A similar sequence type-associated pattern was observed for ST97, which displayed a characteristic accessory gene profile compared with the other sequence types. The newly identified ST9955 formed an independent accessory genome cluster. Although only a limited number of ST398 isolates were included, their accessory gene distribution also differed from that observed in the other sequence types.

### 3.4. Antimicrobial Resistance Phenotypes, Resistance Genes and Resistance Burden

**Figure 5:**
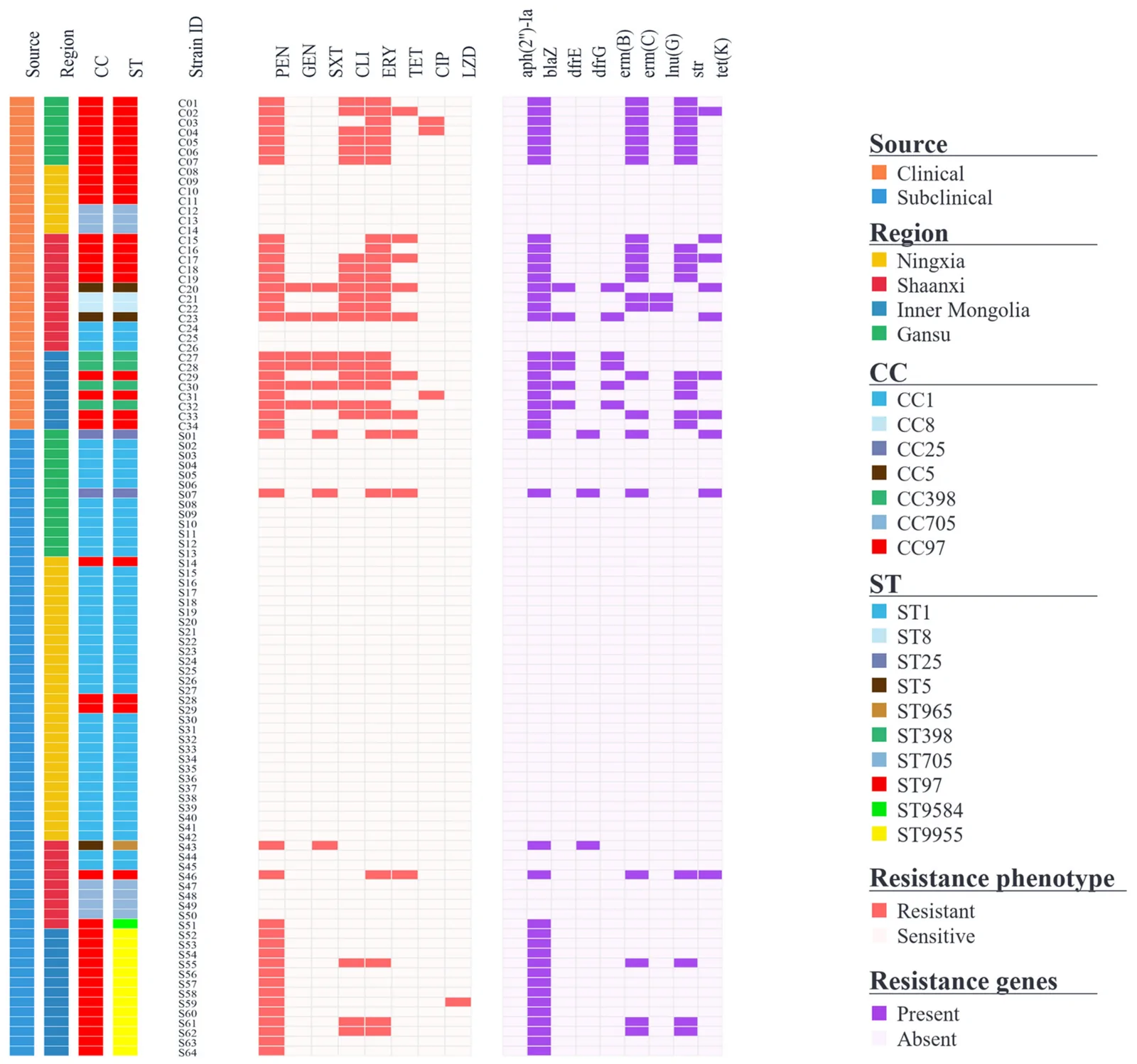
Antimicrobial resistance phenotypes and resistance gene profiles. Rows represent individual isolates, with left-side annotation tracks indicating clinical source, geographic region, clonal complex (CC), sequence type (ST), and isolate ID. The heatmap on the left shows antimicrobial resistance phenotypes, while the heatmap on the right shows the distribution of corresponding resistance genes. Red and pale red cells indicate resistant and susceptible phenotypes, respectively; purple and pale purple cells indicate the presence and absence of resistance genes, respectively. CM, clinical mastitis; SCM, subclinical mastitis; CC, clonal complex; ST, sequence type.

Antimicrobial susceptibility testing showed that resistance to penicillin was the most common phenotype (42.86%, 42/98), followed by erythromycin (28.57%, 28/98), clindamycin (22.45%, 22/98), tetracycline (10.20%, 10/98), trimethoprim–sulfamethoxazole (9.18%, 9/98), gentamicin (6.12%, 6/98), ciprofloxacin (3.06%, 3/98), and linezolid (1.02%, 1/98) (Table 3; Figure 5). All isolates were susceptible to vancomycin, cefoxitin, amikacin, oxacillin, chloramphenicol, and rifampicin, and no isolate met the phenotypic criteria for methicillin resistance.

Resistance gene profiling identified *blaZ* as the most frequently detected determinant (42.86%, 42/98), consistent with the observed penicillin-resistant phenotype (Table 3; Figure 5). Macrolide- and lincosamide-associated genes, including *erm(B)*, *erm(C)*, and *lnu(G)*, were detected among erythromycin- and clindamycin-resistant isolates. The tetracycline resistance determinant *tet(K)* was detected in 10.20% (10/98) of isolates, whereas *dfrE* and *dfrG*, associated with trimethoprim–sulfamethoxazole resistance, were identified in 9.18% (9/98). No acquired resistance determinants corresponding to ciprofloxacin or linezolid resistance were identified within the screening panel used in this study (Table 3; Figure 5). CM-derived isolates showed higher resistance rates overall than SCM-derived isolates (Table 3). Resistance to PEN, GEN, ERY, CLI, TET, and CIP was significantly more frequent in CM isolates (*p* < 0.05), whereas no significant differences were observed for SXT or LZD. Resistance determinants including *blaZ*, *aph(2″)-Ia*, *erm(B)*, *erm(C)*, *lnu(G)*, and *tet(K)* were also more commonly detected in CM-derived isolates.

**Figure 6:**
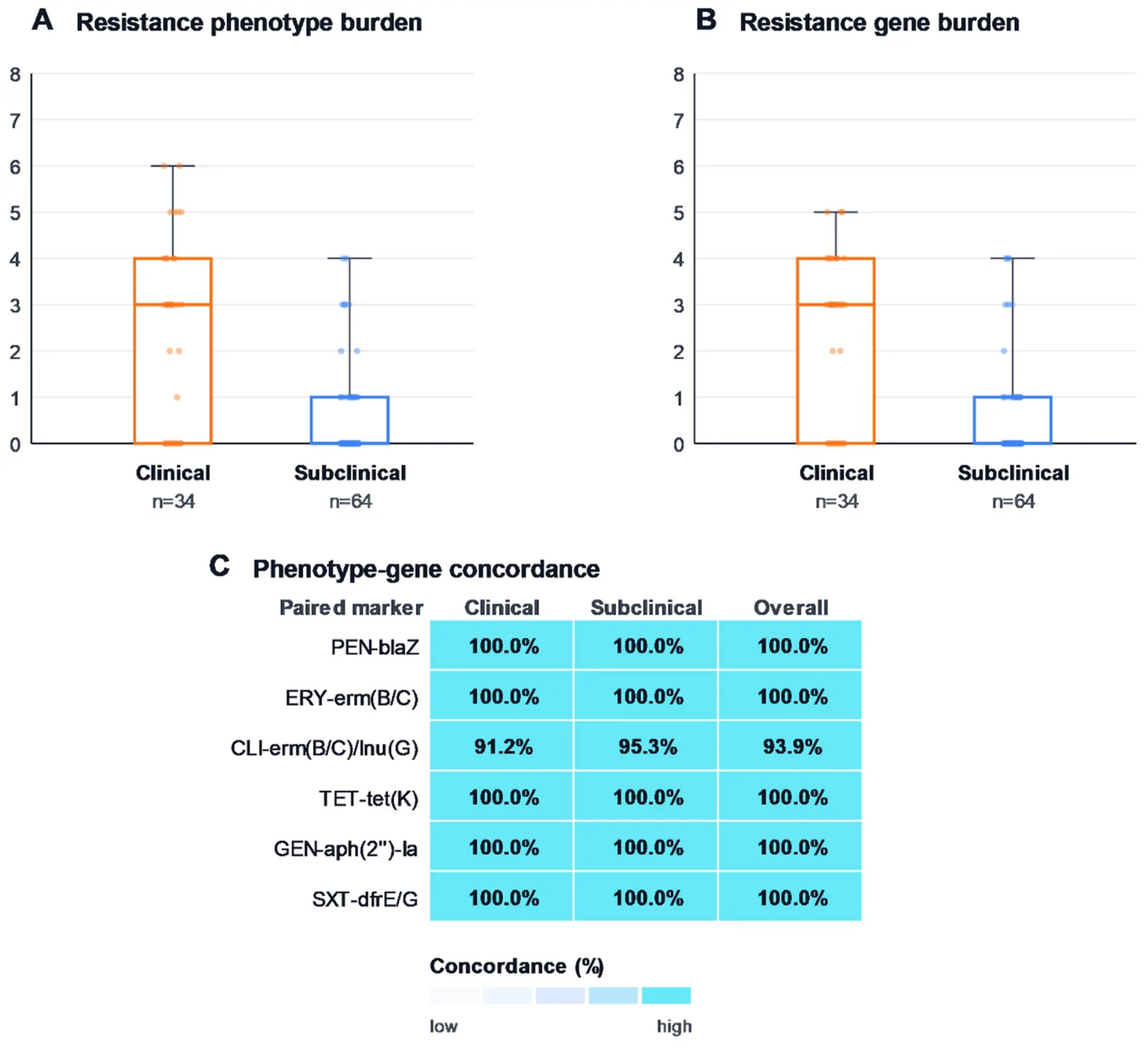
Antimicrobial resistance burden and phenotype–genotype concordance in clinical and subclinical *S. aureus* isolates. (**A**) Phenotypic resistance burden, defined as the number of antimicrobial agents/classes to which each isolate was resistant. (**B**) Resistance gene burden, defined as the number of antimicrobial resistance genes detected in each isolate. Each point represents one isolate, and boxes indicate the interquartile range with the median. (**C**) Concordance between resistance phenotypes and corresponding resistance genes for paired phenotype–gene markers. Concordance was calculated as the percentage of isolates showing matched phenotype and genotype results, including resistant/gene-positive and susceptible/gene-negative combinations.

Analysis of cumulative resistance profiles showed that CM-derived isolates carried a greater resistance burden than SCM-derived isolates (Figure 6A). The same trend was observed for resistance gene burden (Figure 6B), with CM-derived isolates harboring a higher number of resistance determinants overall. Genotype–phenotype concordance was high for most antimicrobial agents (Figure 6C). Complete agreement was observed for PEN–*blaZ*, ERY–*erm(B/C)*, TET–*tet(K)*, GEN–*aph(2″)-Ia*, and SXT–*dfrE/G*, whereas concordance for CLI–*erm(B/C)/lnu(G)* was 93.9%.

### 3.5. Biofilm Formation and Biofilm-Associated Gene Profiles

**Figure 7:**
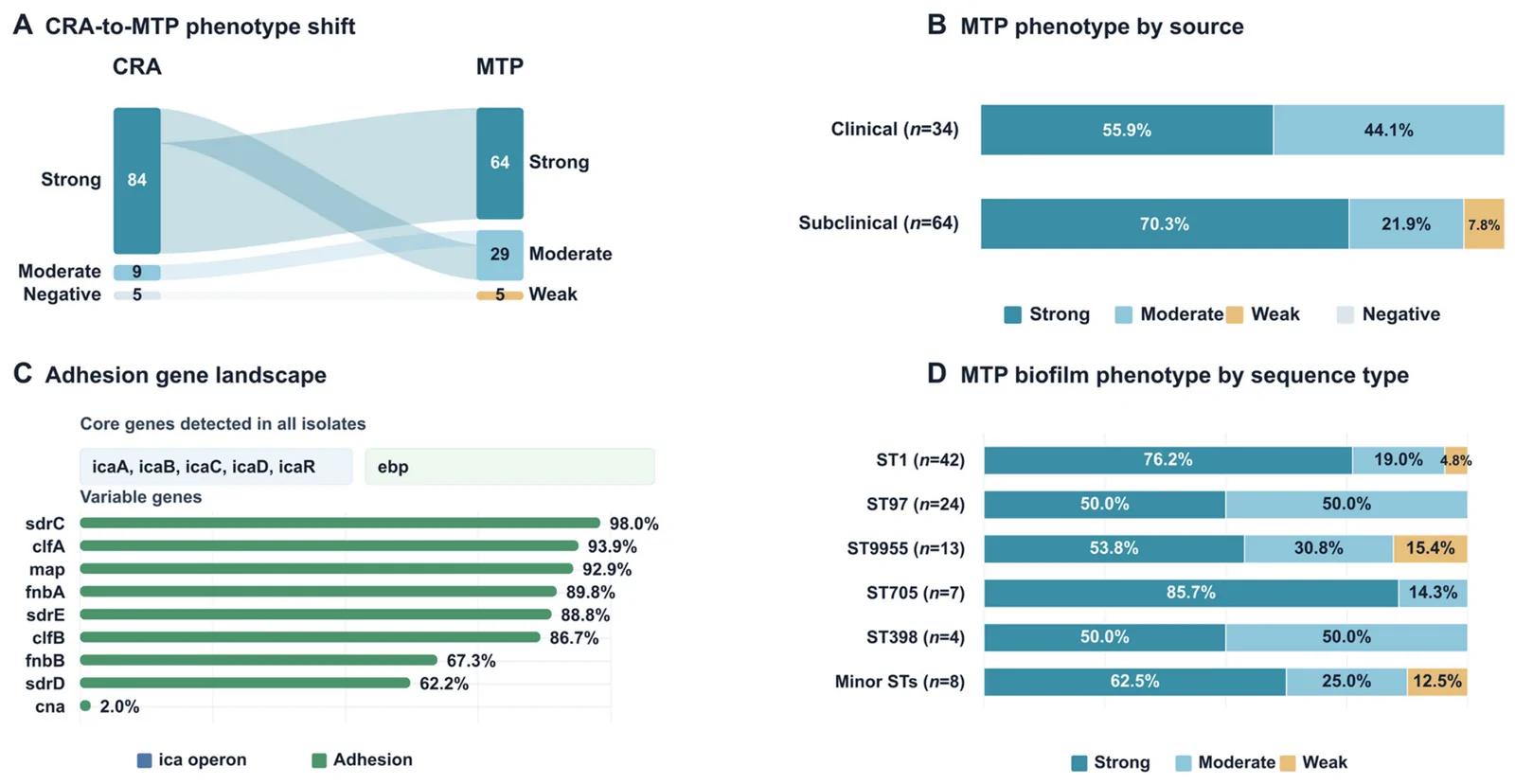
Biofilm formation and adhesion-associated gene profiles of bovine mastitis-derived *S. aureus isolates*. (**A**) Comparison of biofilm phenotypes determined by the CRA and MTP assays. (**B**) Distribution of MTP-defined biofilm phenotypes among isolates from clinical and subclinical mastitis. (**C**) Prevalence of representative biofilm- and adhesion-associated genes. (**D**) Distribution of MTP-defined biofilm phenotypes across the predominant sequence types; Minor STs comprise sequence types represented by few isolates.

Biofilm formation phenotypes of the 98 *S. aureus* isolates are presented in Figure 7A. In the Congo red agar (CRA) assay, 84 isolates (85.7%) were identified as strong biofilm producers, 9 (9.2%) as moderate producers, and 5 (5.1%) as non-producers. In the microtiter plate (MTP) assay, 64 isolates (65.3%) were categorized as strong biofilm producers, 29 (29.6%) as moderate producers, and 5 (5.1%) as weak producers, with no non-producers detected. The two methods were generally consistent in identifying strongly biofilm-forming isolates but differed in the classification of intermediate and weak phenotypes.

MTP phenotype distribution differed significantly between CM- and SCM-derived isolates (*p* = 0.029; Figure 7B). Among CM-derived isolates, 55.9% and 44.1% were classified as strong and moderate biofilm producers, respectively. In contrast, SCM-derived isolates showed a higher proportion of strong biofilm producers (70.3%), followed by moderate (21.9%) and weak (7.8%) phenotypes.

The distribution of biofilm- and adhesion-associated genes is shown in Figure 7C. The *icaA*, *icaB*, *icaC*, *icaD*, *icaR*, and *ebp* genes were detected in all isolates. Among the remaining adhesion-associated genes, detection rates were 98.0% for *sdrC*, 93.9% for clfA, 92.9% for *map*, 89.8% for *fnbA*, 88.8% for *sdrE*, 86.7% for *clfB*, 67.3% for *fnbB*, 62.2% for *sdrD*, and 2.0% for *cna*.

Biofilm phenotypes also varied across sequence types (Figure 7D). ST705 and ST1 showed the highest proportions of strong biofilm producers, at 85.7% and 76.2%, respectively. ST9955 isolates were mainly classified as strong (53.8%) or moderate (30.8%) biofilm producers, whereas ST97 and ST398 each showed equal proportions of strong and moderate phenotypes. Among minor sequence types, strong, moderate, and weak phenotypes accounted for 62.5%, 25.0%, and 12.5%, respectively.

### 3.6. Lineage-Associated Distribution of Virulence Genes

**Figure 8:**
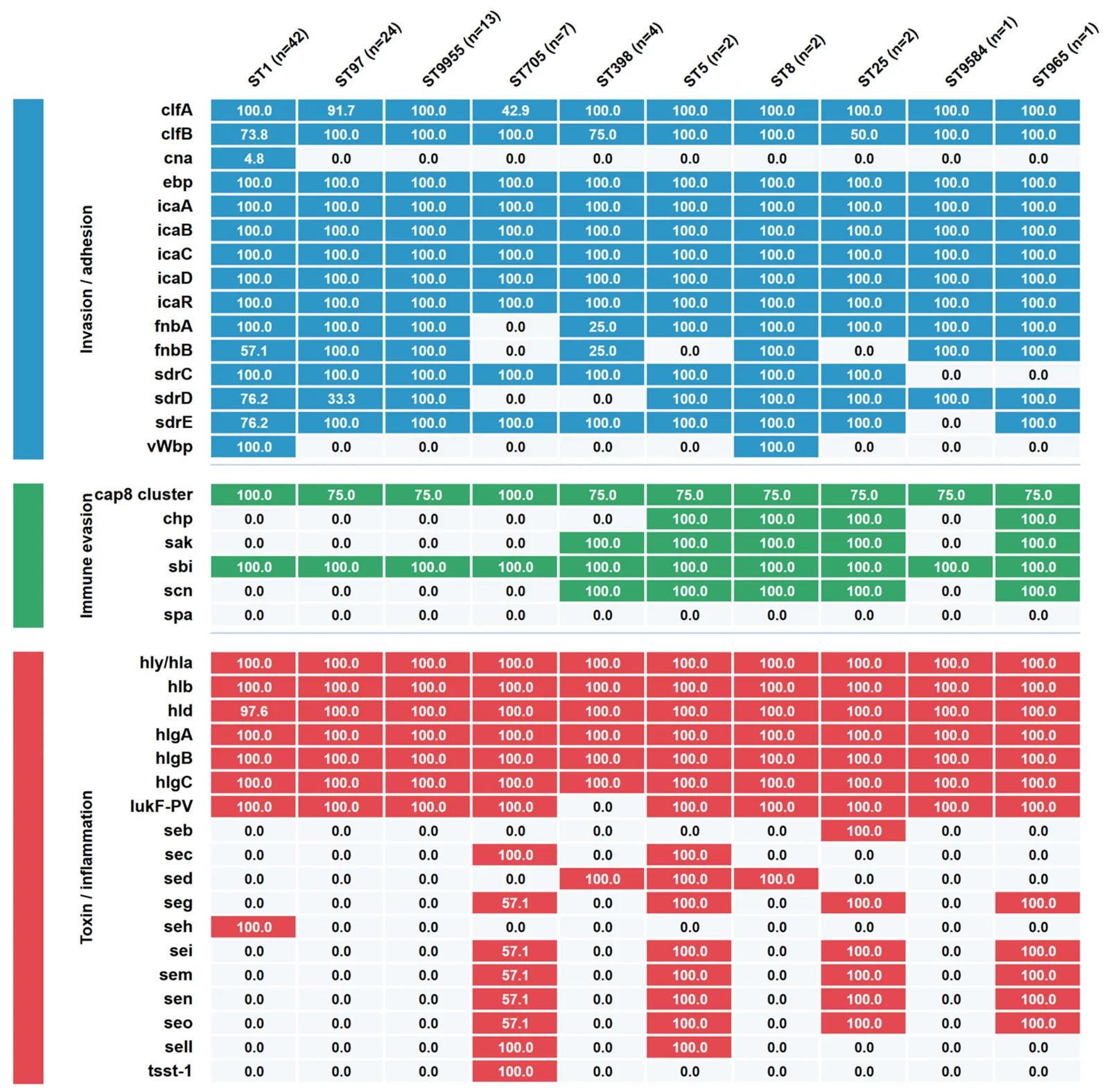
Lineage-associated distribution of virulence-associated gene modules among major sequence types. The prevalence of major virulence-associated genes across different *S. aureus* sequence types (STs). Columns represent STs, with the number of isolates shown in parentheses. Rows represent virulence-associated genes grouped into three functional categories: invasion/adhesion, immune evasion, and toxin/inflammation. Cell values indicate the percentage of isolates within each ST carrying the corresponding gene. Color intensity reflects gene prevalence within each ST.

Distinct differences in virulence gene composition were observed among the *S. aureus* sequence types (Figure 8). Overall, adhesion- and invasion-associated genes were largely conserved across the major lineages, whereas immune evasion and toxin-related genes exhibited greater heterogeneity. Among adhesion- and invasion-associated genes, *icaA*, *icaB*, *icaC*, *icaD*, *icaR*, and *ebp* were detected in all sequence types, while *clfA* and *clfB* were also widely distributed. In contrast, cna was detected only sporadically in ST1. Variation was more apparent for *fnbA*, *fnbB*, *sdrD*, and *vWbp*, with ST705 lacking *fnbA*, *fnbB* and *vWbp*.

Immune evasion genes showed clear lineage-associated variation. The cap8 gene cluster was commonly detected in all major lineages, with 100% prevalence in ST1 and ST705 and 75.0% in most other groups. The *sbi* gene was universally present, whereas *chp*, *sak* and *scn* were restricted mainly to ST5, ST8, ST25, and ST965 isolates, and were absent from ST1, ST97, ST9955, and ST705.

Toxin- and inflammation-associated genes also showed marked lineage-specific distribution. Hemolysin-related genes, including *hla*, *hlb*, *hld*, *hlgA*, *hlgB* and *hlgC*, were highly conserved across most sequence types. *lukF-PV* was detected in multiple lineages but was absent from ST398. Among enterotoxin- and toxic shock-associated genes, *seh* was confined to ST1; *sec*, *sell* and *tsst-1* were mainly detected in ST705; *sed* was detected in ST398, ST5, and ST8; and *seb* was found only in ST25. In addition, *seg*, *sei*, *sem*, *sen* and *seo* showed a clustered distribution, being detected in ST705, ST5, ST25, and ST965, with partial prevalence in ST705.

### 3.7. Co-Occurrence Patterns of Virulence-Associated Genes

**Figure 9:**
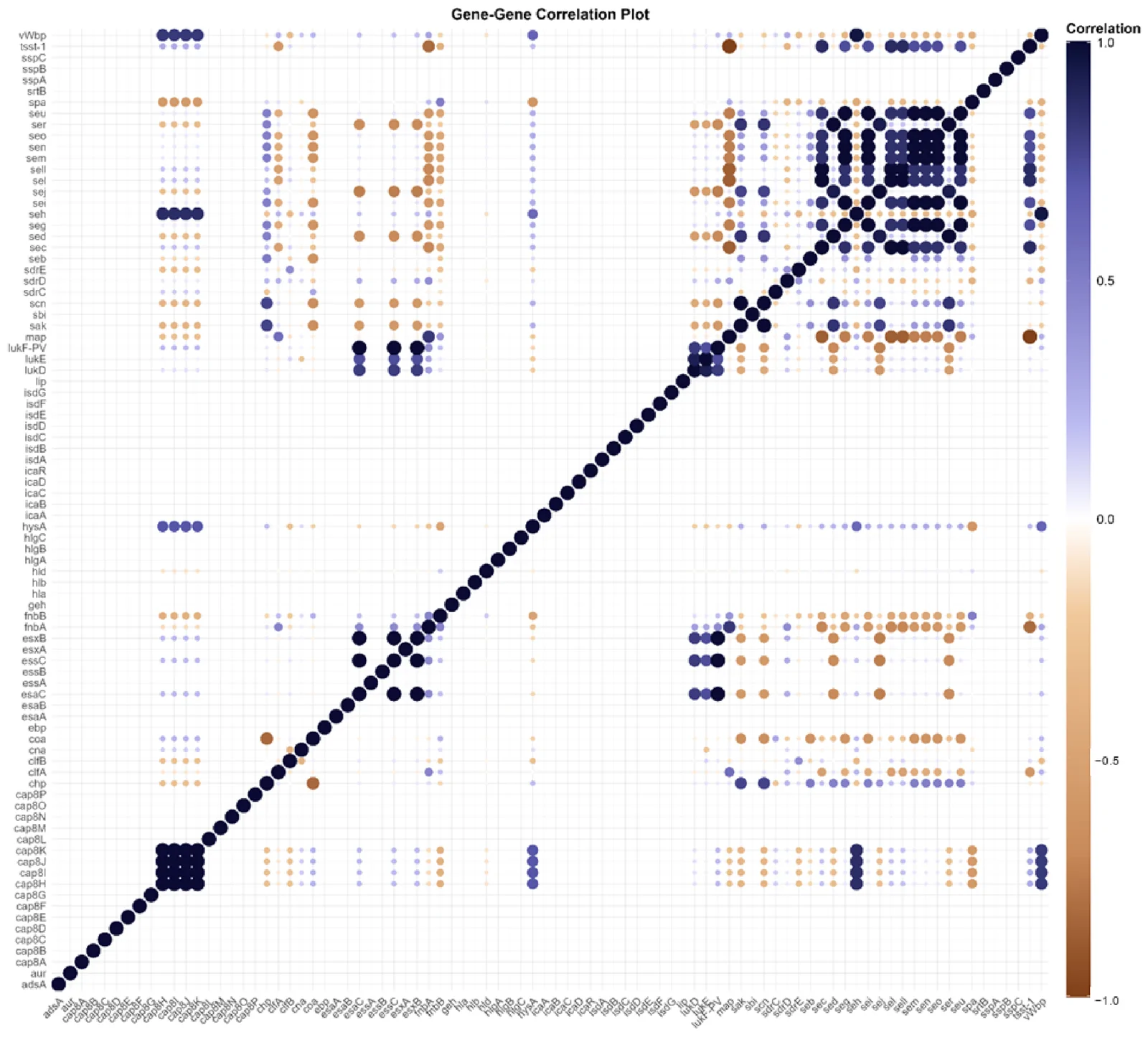
Pairwise correlation matrix of virulence-associated genes in *S. aureus* isolates. Each circle represents the correlation between two virulence genes based on their presence or absence across the isolate collection. Circle color indicates the direction and magnitude of the correlation coefficient, with blue denoting positive correlations and brown denoting negative correlations. Circle size is proportional to the absolute value of the correlation coefficient. The diagonal represents self-correlations.

Gene-gene correlation analysis indicated that virulence-associated genes in the *S. aureus* isolates were non-randomly distributed and formed several distinct co-occurrence modules (Figure 9). The most prominent cluster involved the capsular polysaccharide synthesis genes (*cap8A–cap8P*), which exhibited strong positive correlations. A similar pattern was observed for the biofilm-associated ica operon genes (*icaA–icaD* and *icaR*), reflecting their coordinated distribution across the isolates. T7SS-related genes (*esaA*, *esaB*, *esaC*, *essA*, *essB*, *essC*, *esxA* and *esxB*) also formed a tightly correlated cluster, whereas the iron-regulated surface determinant (*isd*) genes showed weaker but still coordinated correlation patterns.

Enterotoxin-associated genes displayed a distinct clustered distribution. In particular, *seg*, *sei*, *sem*, *sen* and *seo* formed a tightly correlated group consistent with the classical enterotoxin gene cluster (*egc*). In contrast, other enterotoxin genes, including *seb*, *sec*, *sed*, *seh* and *sell*, showed more dispersed correlation patterns, indicating greater heterogeneity in their distribution among isolates.

Among immune evasion-associated genes, *chp*, *sak*, and *scn* showed clear co-occurrence, whereas *sbi* displayed a broader distribution pattern without forming a similarly tight cluster. By comparison, adhesion- and invasion-associated genes, including *clfA*, *clfB*, *fnbA*, *fnbB*, *sdrC*, *sdrD* and *sdrE*, exhibited more heterogeneous correlation patterns and did not form a clearly defined module.

## 4. Discussion

Core-genome SNP analysis, MLST, and spa typing showed considerable diversity among the bovine mastitis-associated *S. aureus* isolates recovered in this study, including multiple clonal complexes, sequence types, and spa types. These results indicate that the *S. aureus* population circulating in northwestern China is composed of several coexisting lineages rather than a single dominant epidemic clone. Similar population structures have been reported in bovine-associated *S. aureus* from different geographic regions, where a small number of dominant lineages coexist with multiple sporadic clones [16,38]. Despite this diversity, ST1 and ST97 together accounted for more than two-thirds of all isolates, indicating that these two lineages predominate in this region. The predominance of ST1 and ST97 may reflect their successful adaptation and persistence in bovine populations [14,15,39]. Cattle movement and herd replacement practices may also contribute to the dissemination of these lineages among farms, although this could not be confirmed due to the lack of animal movement data. CC97/ST97 is recognized as one of the major bovine-associated *S. aureus* lineages worldwide and has been repeatedly identified in mastitis cases from Europe, North America, and Asia [13,15,16,19]. In contrast, although CC1/ST1 has historically been more common in human-associated *S. aureus* populations, recent studies from China and New Zealand have increasingly reported this lineage in bovine mastitis isolates [14,19], indicating increasing adaptation to the bovine host.

The two dominant lineages exhibited contrasting distributions between clinical and subclinical mastitis cases. ST97 was more frequently recovered from CM cases, whereas ST1 predominated among SCM isolates. Previous studies have consistently identified CC97 as a major bovine-associated lineage and a common cause of intramammary infection in dairy cattle [13,15,16]. The predominance of ST97 among CM isolates in the present study therefore supports its continued epidemiological importance in bovine mastitis. In contrast, ST1 was mainly recovered from SCM cases. Subclinical *S. aureus* mastitis is typically characterized by prolonged intramammary colonization and sustained within-herd transmission, often in the absence of obvious clinical signs [2,16,40]. Although persistence was not directly evaluated in the present study, the predominance of ST1 among SCM isolates may be consistent with epidemiological characteristics that facilitate long-term circulation within dairy herds. Further comparative genomic and phenotypic studies are needed to determine whether lineage-specific factors contribute to the distinct distribution patterns observed between ST1 and ST97.

A novel sequence type, ST9955, was identified exclusively among subclinical mastitis isolates from Inner Mongolia. Phylogenetic analysis placed ST9955 within the CC97-associated lineage, whereas accessory genome analysis revealed a distinct genomic composition, suggesting diversification despite its close evolutionary relationship with ST97. Previous genomic studies have shown that bovine mastitis-associated *S. aureus* populations are often structured by lineage and geography, with successful clones persisting and diversifying within specific regions or dairy production systems [14,39,41]. The restricted distribution of ST9955 within a single geographic region is therefore consistent with local expansion or regional diversification of a CC97-related lineage. The epidemiological significance of ST9955 remains uncertain because of the limited number of isolates and their restricted geographic origin. Nevertheless, its repeated recovery from subclinical mastitis cases warrants further attention. Whether this distribution pattern reflects lineage-associated biological characteristics or regional epidemiological factors remains unclear. Further surveillance and comparative genomic analyses will be required to clarify the origin, dissemination, and potential significance of this lineage in bovine mastitis.

Accessory genome analysis revealed marked lineage-specific gene distribution patterns among the major sequence types. Previous studies have shown that variation in accessory gene content, particularly within mobile genetic elements, contributes substantially to lineage diversification and host adaptation in *S. aureus* [15,16,42]. Consistent with this, ST1, ST97, ST9955, and ST398 formed distinct accessory genome clusters, indicating that population differentiation in bovine mastitis-associated *S. aureus* is shaped by variation in both the core and accessory genomes. Despite their close phylogenetic relationship within CC97, ST9955 and ST97 displayed clearly distinct accessory genome profiles. Similar patterns have been reported in bovine-associated *S. aureus*, where closely related lineages may exhibit distinct accessory genome compositions despite sharing a common phylogenetic background, reflecting the dynamic acquisition and loss of accessory genetic elements [16,17]. ST398 also exhibited a distinct accessory genome profile and was detected exclusively among clinical mastitis isolates. Although only a small number of ST398 isolates were identified, this observation is noteworthy because ST398 is a recognized livestock-associated lineage with documented potential for antimicrobial resistance acquisition and interspecies transmission [15,43]. These findings further support the contribution of accessory genome variation to the diversification of bovine mastitis-associated *S. aureus* lineages.

Methicillin-resistant *S. aureus* (MRSA), particularly livestock-associated MRSA (LA-MRSA), is widely recognized as a One Health issue due to its zoonotic relevance and global spread. Distinct distribution patterns have been reported among major LA-MRSA lineages, with ST9 mainly identified in Asia and ST398 predominantly in Europe and North America [15,43]. In this study, no MRSA was detected, and all isolates remained susceptible to cefoxitin and oxacillin, indicating the absence of detectable methicillin resistance in bovine mastitis-associated *S. aureus* from this region. ST398 was identified at low frequency and only in clinical mastitis cases; these isolates lacked *mec*-mediated resistance but carried other acquired resistance genes, suggesting that this lineage persists locally as a livestock-associated MSSA clone without evidence of MRSA evolution.

Resistance to commonly used antimicrobials, particularly penicillin, erythromycin, and clindamycin, was frequently observed. CM isolates showed a higher resistance burden than SCM isolates, consistent with previous reports describing broader resistance profiles in clinical mastitis cases, which may be associated with increased antimicrobial exposure [44,45]. However, detailed treatment histories and quantitative antimicrobial consumption data were unavailable, limiting causal interpretation of the association between antimicrobial exposure and resistance patterns. Farm-level information indicated the use of several antimicrobial classes, including penicillins, cephalosporins, aminoglycosides, and lincosamides, but their specific contribution to resistance selection requires further investigation. Overall, resistance phenotypes were largely explained by known genes, including *blaZ*, *erm(B/C)*, *tet(K)*, *aph(2″)-Ia* and *dfr* determinants, showing a high degree of concordance between genotype and phenotype. This pattern suggests that antimicrobial resistance in the present population is mainly driven by dissemination of established resistance elements rather than emergence of novel mechanisms [46].

Biofilm formation is widely recognized as a key factor associated with persistent intramammary infection and reduced therapeutic efficacy in *S. aureus* mastitis [47,48]. In the present study, most isolates exhibited moderate to strong biofilm-forming ability, which was consistent with the widespread distribution of major biofilm- and adhesion-associated genes, including *icaA–D/icaR*, *clfA*, *clfB* and *fnbA/fnbB.* These determinants encode functional factors involved in bacterial adhesion, surface colonization, and extracellular matrix formation, providing a genetic basis for the biofilm phenotypes observed in this study [49,50,51,52,53]. However, biofilm formation is a complex trait influenced by bacterial background, regulatory networks, and environmental conditions; therefore, individual biofilm-associated genes alone cannot fully explain phenotypic variation [49]. Notably, stronger biofilm-forming phenotypes were more frequently observed among subclinical mastitis isolates than among clinical mastitis isolates, with significant differences in MTP distribution between the two groups. Although biofilm formation enhances bacterial adhesion and colonization, biofilm-associated traits have also been linked to persistent infection in bovine intramammary *S. aureus* isolates [48,50]. Therefore, the enrichment of strong biofilm-forming isolates among subclinical cases in this study may reflect persistence-associated phenotypes during prolonged intramammary infection rather than increased ability to cause overt clinical mastitis [47,51]. Nevertheless, CRA and MTP assays are in vitro methods that cannot fully reproduce the complex intramammary environment, and further validation using in vivo models and longitudinal studies is required [50].

Distinct virulence gene repertoires were observed among different *S. aureus* sequence types. Overall, genes associated with adhesion and biofilm formation were highly conserved across most lineages, particularly *icaA-D*, *icaR*, *ebpS*, *clfA* and *clfB*. The *ica* operon is responsible for the synthesis of polysaccharide intercellular adhesin/poly-N-acetylglucosamine (PIA/PNAG), a key component of biofilm formation [51,52,53], whereas members of the *clf*, *fnb* and *sdr* families encode surface-associated adhesins that mediate attachment to host tissues and extracellular matrix components, thereby contributing to mammary colonization and persistent infection [5]. Correlation analysis further revealed stable co-occurrence patterns among *icaA-D*, *icaR* and the *cap8A-P* capsule biosynthesis cluster, indicating that biofilm- and capsule-associated determinants tend to be maintained as coordinated functional units. Since capsule production enhances resistance to phagocytic clearance and biofilm formation promotes persistence under host immune and antimicrobial pressures, both traits have been linked to chronic intramammary infections and long-term carriage [10,54]. Consistent with this observation, ST1, ST97, and the newly identified ST9955 harbored relatively complete sets of adhesion-associated genes, while ST1 additionally exhibited strong biofilm-forming capacity, suggesting enhanced adaptation to mammary colonization and long-term persistence [50].

Immune evasion genes exhibited a more pronounced lineage-dependent distribution. Although the *cap8* cluster and *sbi* were widely distributed among most lineages, *chp*, *sak* and *scn* were mainly restricted to ST5, ST8, ST25, and ST965, and were largely absent from the dominant lineages ST1, ST97, ST9955, ST705 and ST9584. These three genes also formed a tightly linked co-occurrence cluster in the correlation network, consistent with their organization within the immune evasion cluster (IEC). The IEC is typically carried by β-hemolysin-converting bacteriophages and encodes immune-modulating factors, including the staphylococcal complement inhibitor, chemotaxis inhibitory protein, and staphylokinase [55]. Previous studies have shown that IEC genes are common among human-adapted *S. aureus* lineages but are frequently absent from bovine-adapted lineages, particularly CC97. Reacquisition of the IEC has been proposed as a hallmark of bovine-to-human host adaptation in CC97, highlighting its importance in host transition and human adaptation [43,56].

Toxin-associated genes likewise exhibited a modular organization. While hemolysin genes, including *hla*, *hlb*, *hld*, and *hlgABC*, were highly conserved across nearly all STs, superantigen genes showed marked lineage-specific distributions. For example, *seh* was predominantly associated with ST1, whereas *sec*, *sell* and *tst* were largely confined to ST705. These genes are frequently linked to staphylococcal pathogenicity islands (SaPIs), and their distribution is strongly influenced by horizontal gene transfer [57,58]. In addition, *seg*, *sei*, *sem*, *sen* and *seo* formed the strongest co-occurrence cluster in the correlation analysis. These genes constitute the classical enterotoxin gene cluster (egc), which is generally maintained as an intact genetic unit and has been described as an important reservoir of superantigen genes in *S. aureus* [59]. The observed lineage-specific distribution of superantigen genes is therefore consistent with the contribution of mobile genetic elements to shaping toxin gene repertoires in different clonal backgrounds.

Beyond classical virulence determinants, genes associated with the type VII secretion system (T7SS) and the iron-regulated surface determinant (Isd) system also exhibited clear co-occurrence patterns. T7SS has been implicated in interbacterial competition, host adaptation, and virulence regulation [60,61], whereas the Isd system plays a central role in heme-iron acquisition under iron-limited host conditions [62]. The coordinated distribution of these loci further suggests that successful adaptation of bovine-associated *S. aureus* involves multiple complementary functional systems rather than individual virulence factors acting in isolation.

These findings support the existence of lineage-associated virulence modules among bovine mastitis-associated *S. aureus.* ST1, ST97, and ST9955 predominantly retained functional traits related to adhesion, biofilm formation, and persistence, whereas ST705 and several minor lineages carried a broader repertoire of immune evasion or superantigen-associated genes. Because ST5, ST8, ST25, ST9584, and ST965 were represented by only a limited number of isolates, the lineage-specific characteristics observed for these groups should be interpreted cautiously. Further transcriptomic, proteomic, and infection-model studies will be required to determine whether these genetic differences translate into distinct colonization, immune evasion, or inflammatory phenotypes.

## 5. Conclusions

This study comprehensively characterized the population structure, antimicrobial resistance profiles, biofilm-forming capacity, and virulence gene repertoire of bovine mastitis-associated *S. aureus* from large-scale dairy farms in northwestern China. Despite considerable genetic diversity, the population was dominated by a few successful lineages, particularly ST1 and ST97, which exhibited distinct epidemiological and pathogenic features. ST1 was enriched among subclinical mastitis isolates and exhibited strong biofilm-forming ability, whereas ST97/CC97 was more frequently detected among clinical mastitis isolates in the present collection. A novel sequence type, ST9955, was identified exclusively among subclinical mastitis isolates from Inner Mongolia, suggesting a potentially region-associated lineage within the present collection. Although no MRSA was detected, the higher resistance burden observed in clinical isolates highlights the continued importance of antimicrobial stewardship in dairy production. Overall, the findings indicate that the pathogenic potential of bovine *S. aureus* is more closely linked to lineage-specific combinations of adhesion, biofilm, immune evasion, and toxin-associated traits than to individual virulence factors. These results provide new insights into the population biology and host adaptation of bovine *S. aureus* and support the development of targeted surveillance and control strategies within a One Health framework.

## Figures and Tables

**Table 1:** Prevalence of *S. aureus* in milk samples from clinical and subclinical mastitis in different regions.

Region	Clinical Mastitis			Subclinical Mastitis			Total		
	No. of Samples	Positive Samples	Prevalence (%)	No. of Samples	Positive Samples	Prevalence (%)	Total Samples	Total Positive	Total Prevalence (%)
Gansu	64	7	10.94	183	13	7.10	247	20	8.10
Ningxia	50	7	14.00	249	29	11.65	299	36	12.04
Inner Mongolia	66	8	12.12	138	13	9.42	204	21	10.29
Shaanxi	87	12	13.79	105	9	8.57	192	21	10.94
Total	267	34	12.73	675	64	9.48	942	98	10.40

Prevalence was calculated as positive samples/total samples. Differences between mastitis types and among regions were assessed using Pearson’s chi-square test; all comparisons were not statistically significant (*p* > 0.05).

**Table 2:** Sequence types showing significantly different distributions between clinical mastitis and subclinical mastitis isolates.

ST	Total Isolates, n (%)	SCM, n (%)	CM, n (%)	*p*-Value
ST1	42 (42.86)	39 (60.94)	3 (8.82)	<0.001
ST97	24 (24.49)	4 (6.25)	20 (58.82)	<0.001
ST9955	13 (13.27)	13 (20.31)	0 (0.00)	0.004
ST398	4 (4.08)	0 (0.00)	4 (11.76)	0.013

ST, sequence type; CM, clinical mastitis; SCM, subclinical mastitis. Percentages were calculated using the total number of isolates in each group as the denominator. Differences between CM and SCM isolates were assessed using Fisher’s exact test. Only STs with significant differences are shown.

**Table 3:** Antimicrobial resistance phenotypes and associated resistance genes among bovine mastitis-associated *S. aureus* isolates stratified by clinical source.

Antimicrobial Class	Antimicrobial Agent	Associated Resistance Gene (s)	Overall Phenotype, n/N (%)	Overall Gene, n/N (%)	CM Phenotype, n/N (%)	CM Gene, n/N (%)	SCM Phenotype, n/N (%)	SCM Gene, n/N (%)	Phenotype *p*-Value	Gene *p*-Value
Beta-lactam	PEN	*blaZ*	42/98 (42.86%)	42/98 (42.86%)	24/34 (70.59%)	24/34 (70.59%)	18/64 (28.13%)	18/64 (28.13%)	<0.001	<0.001
Aminoglycoside	GEN	*aph(2″)-Ia*	6/98 (6.12%)	6/98 (6.12%)	6/34 (17.65%)	6/34 (17.65%)	0/64 (0.00%)	0/64 (0.00%)	0.001	0.001
Folate pathway inhibitor	SXT	*dfrE/dfrG*	9/98 (9.18%)	9/98 (9.18%)	6/34 (17.65%)	6/34 (17.65%)	3/64 (4.69%)	3/64 (4.69%)	0.061	0.061
Macrolide	ERY	*erm(B)/erm(C)*	28/98 (28.57%)	28/98 (28.57%)	22/34 (64.71%)	22/34 (64.71%)	6/64 (9.38%)	6/64 (9.38%)	<0.001	<0.001
Lincosamide	CLI	*erm(B)/erm(C)/lnu(G)*	22/98 (22.45%)	28/98 (28.57%)	19/34 (55.88%)	22/34 (64.71%)	3/64 (4.69%)	6/64 (9.38%)	<0.001	<0.001
Tetracycline	TET	*tet(K)*	10/98 (10.20%)	10/98 (10.20%)	7/34 (20.59%)	7/34 (20.59%)	3/64 (4.69%)	3/64 (4.69%)	0.030	0.030
Fluoroquinolone	CIP	Not detected in this matrix	3/98 (3.06%)	NA	3/34 (8.82%)	NA	0/64 (0.00%)	NA	0.039	NA
Oxazolidinone	LZD	Not detected in this matrix	1/98 (1.02%)	NA	0/34 (0.00%)	NA	1/64 (1.56%)	NA	1.000	NA

Values are shown as n/N (%). CM, clinical mastitis; SCM, subclinical mastitis; NA, not applicable. Gene prevalence indicates the proportion of isolates carrying at least one associated resistance gene. Differences between CM and SCM isolates were assessed using Fisher’s exact test, with *p* < 0.05 considered statistically significant.

## Data Availability

Data will be made available on request.

## Abbreviations

The following abbreviations are used in this manuscript:

STs	sequence types
CCs	clonal complexes
SCM	subclinical mastitis
CM	clinical mastitis
MALDI-TOF MS	matrix-assisted laser desorption/ionization time-of-flight mass spectrometry
MLST	multilocus sequence typing
VFDB	Virulence Factor Database
PEN	penicillin
FOX	cefoxitin
OXA	oxacillin
TET	tetracycline
AMK	amikacin
TMP–SMX	trimethoprim–sulfamethoxazole
GEN	gentamicin
LZD	linezolid
CHL	chloramphenicol
CLI	clindamycin
ERY	erythromycin
CIP	ciprofloxacin
RIF	rifampicin
VAN	vancomycin
MIC	minimum inhibitory concentration
CLSI	Clinical and Laboratory Standards Institute
MTP	microtiter plate
PIA/PNAG	polysaccharide intercellular adhesin/poly-N-acetylglucosamine
IEC	immune evasion cluster
T7SS	the type VII secretion system
